# Death from Chloroform, in San Francisco

**Published:** 1864-11

**Authors:** 


Death from Chloroform, in San Francisco.—Mrs. ———, a native of California, of highly nervous temperament, was placed in the operating chair to have a tooth extracted. She was in great dread of the instruments, and while much excited from this cause, the chloroform was administered, by holding the napkin above and near the nostrils. She had some difficulty in managing the respiration, and, after inhaling it imperfectly a short time, complained that she could not take it, and seized the hand of the operator, removing it from her face. Without using the chloroform any longer, her hands were held by an assistant, and the tooth extracted without difficulty. Immediately after the withdrawal of the forceps from her mouth, her jaws were clenched and her head thrown back, by spasmodic contraction of the muscles. The breathing was arrested, the face grew livid, and death rapidly ensued. The immediate cause of death appears to have been apnœa, from the spasmodic closure of the glottis or larynx. The masseter, respiration, and spinal muscles, and also the flexors of the extremities, continued in a state of tonic spasm, till relaxed by death. We are satisfied that there was no mismanagement of the chloroform in this case—unless to use it at all be mismanagement. There is quite as much reason to allege that it was not pushed far enough, as that it was pushed too far. Possibly a longer inhalation would have carried the patient safely beyond the range of spasm, and into the tranquil region of anæsthesia. There was no disease of the heart or other organs to discourage the use of the agent.—*San Francisco Med. Press.*

